# Youth’s social network structures and peer influences: study protocol MyMovez project – Phase I

**DOI:** 10.1186/s12889-018-5353-5

**Published:** 2018-04-16

**Authors:** Kirsten E. Bevelander, Crystal R. Smit, Thabo J. van Woudenberg, Laura Buijs, William J. Burk, Moniek Buijzen

**Affiliations:** Behavioural Science Institute, Radboud University, Communication Science, P.O. Box 9104, 6500 HE Nijmegen, The Netherlands

**Keywords:** Social network, Peer influence, Social influence agent, Dietary intake, Physical activity, Social media, Social network intervention, Smartphone, Activity tracker, Research app

## Abstract

**Background:**

Youth are an important target group for social network interventions, because they are particularly susceptible to the adaptation of healthy and unhealthy habits and behaviors of others. They are surrounded by ‘social influence agents’ (i.e., role models such as family, friends and peers) that co-determine their dietary intake and physical activity. However, there is a lack of systematic and comprehensive research on the implementation of a social network approach in health campaigns. The *MyMovez* research project aims to fill this gap by developing a method for effective social network campaign implementation. This protocol paper describes the design and methods of Phase I of the *MyMovez* project, aiming to unravel youth’s social network structures in combination with individual, psychosocial, and environmental factors related to energy intake and expenditure. In addition, the *Wearable Lab* is developed to enable an attractive and state-of-the-art way of collecting data and online campaign implementation via social networks.

**Methods:**

Phase I of the *MyMovez* project consists of a large-scale cross-sequential cohort study (*N* = 953; 8-12 and 12-15 y/o). In five waves during a 3-year period (2016-2018), data are collected about youth’s social network exposure, media consumption, socialization experiences, psychological determinants of behavior, physical environment, dietary intake (snacking and drinking behavior) and physical activity using the *Wearable Lab*. The *Wearable Lab* exists of a smartphone-based research application (app) connected to an activity tracking bracelet, that is developed throughout the duration of the project. It generates peer- and self-reported (e.g., sociometric data and surveys) and experience sampling data, social network beacon data, real-time physical activity data (i.e., steps and cycling), location information, photos and chat conversation data from the app’s social media platform *Social Buzz*.

**Discussion:**

The *MyMovez* project - Phase I is an innovative cross-sequential research project that investigates how social influences co-determine youth’s energy intake and expenditure. This project utilizes advanced research technologies (*Wearable Lab*) that provide unique opportunities to better understand the underlying processes that impact youths’ health-related behaviors. The project is theoretically and methodologically pioneering and produces a unique and useful method for successfully implementing and improving health campaigns.

**Electronic supplementary material:**

The online version of this article (10.1186/s12889-018-5353-5) contains supplementary material, which is available to authorized users.

## Background

Most Western societies devote substantial financial and human resources to the development and implementation of media health campaigns. The alarming rise of obesity has led to a particular increase in efforts aimed at the prevention and reduction of child and adolescent obesity. Youth are an important target group for health communication, because they are especially susceptible to environmental influences and because habits formed during this developmental period persist into adulthood. Unfortunately, media health campaigns often have a disappointing impact. Health messages may be successful in changing behaviors in a laboratory setting, but evaluation research consistently shows little impact in society [1].

An explanation for the gap between laboratory campaign design and behavioral adoption in society is that most mass media campaigns do not take into account the social context in which the message is received. The crucial role of the social environment on youth’s weight-related behaviors is increasingly recognized by academics, health practitioners, and politicians [2–5]. Youth are surrounded by family members, peers and other role models (i.e., *social influence agents*) who support and/or undermine the targeted behaviors. Not only do these social influences compete with media influences, they also play a crucial role in how media messages are transmitted, received, and evaluated. Especially peers are of critical importance when youth reach adolescence, and they play a crucial role in message processing [6]. Recent developments in the media landscape, in particular the explosive growth of social media, present a practical way to incorporate the social context in campaign implementation. Aside from advantages such as wide reach, low costs, and ample technological possibilities, social media enables to reach target recipients via their social networks [7].

Social network interventions make use of peer influence to change behavior throughout the social network [8]. Contrasting traditional mass health intervention campaigns in which *all* individuals are exposed to an intervention message, social network campaigns only target *influential* individuals to perform and stimulate specific behaviors. When social influence agents disseminate the appropriate behavior successfully, it is expected that the behavior will be incorporated by others within their social networks. Subsequently, a social network approach has potential to cause long-term behavior change when the behavior becomes incorporated as the group norm. The approach has been successful in improving health-related behaviors such as quitting smoking [9, 10], increasing condom use [11], and promoting water drinking [12]. However, despite their increasing popularity, it remains unclear how social network campaigns actually work and what are the most effective ways to implement them. Greater understanding of social network structures and social influence processes is crucial for the development of theory and evidence-based social network interventions.

The overarching aim of this 5-year research project is to develop and test a method for effective media health campaign implementation by targeting the most powerful influence agents in youth’s social networks. The *MyMovez* project focuses on two important behavior types of the energy balance equation (i.e., energy intake and expenditure): the consumption of snack foods and (sugar-sweetened) beverages and physical activity (PA). In this protocol paper, we describe the design and methods of the first Phase of the *MyMovez* project. Phase I focuses on two parallel trajectories: Part A investigates youth’s social network structures and youths’ positions within these networks; and examines how media, personal and social influences co-determine energy intake and expenditure. Specifically, the overall aim is to i) identify the most powerful influence agents and examine similarity of health behaviors in youth’s social networks, and to ii) investigate which factors determine youths’ network positions as well as their energy intake and expenditure. In Part B, the research technology *Wearable Lab* is developed to enable data collection (and the campaign implementation in Phase II).

During a period of 3 years, a large-scale cross-sequential cohort study is conducted (*N* = 953) along with several focus group studies. The main sample of the *MyMovez* project – Phase I exists of two age-cohorts (8-12 and 12-15 y/o) of children and adolescents at 21 primary and secondary schools throughout the Netherlands. The recruitment of schools and participants took place with the assistance of several Dutch Public Health Services (GGDs). The *MyMovez* project is funded by the European Research Council (ERC) and carried out by a multidisciplinary research team of the Behavioural Science Institute, Radboud University, Nijmegen, in the Netherlands.

### Part A: Unraveling youth’s social network structures and positions

This project uses approaches and theories centered around the influence of the social environment to investigate how social influence agents can be identified, the relative importance of selection and influence processes, and what distinguishes social influence agents compared to other peers (i.e., ‘followers’). Ample empirical studies have proven the strong influence of peers on young people’s consumption behavior and physical activity [13–15]. For example, studies showed that youth follow and model each other’s eating behavior and that peers set guidelines or ‘social norms’ for others’ food choice and intake [16, 17]. In addition, peer groups are found to play a role in shaping physical activity behavior and youth are more active when they are together with peers [15, 18]. Social network interventions integrate peer influence processes in their campaigns to change behavior throughout the social network [8]. Yet, more research is needed to unravel how social influence agents can be identified and selected.

To date no systematic examination has been conducted on the selection process of social influence agents in peer-driven intervention studies. For example, most research on social networks are based on selecting influence agents who receive the most ‘friendship’ nominations; however, it is unclear whether this selection method is justified in the context of energy intake and expenditure [17]. Selecting influence agents based on ‘advice’ or ‘respect’ nominations (or a combination between these questions) [19] might identify other persons holding influential positions in the network compared to friendship queries. Also, these types of questions can be regarded as general and not directed to specific health behaviors, whereas it might be more useful to ask specific nomination questions (e.g., ‘with whom do you talk about PA?’ or ‘who is modeled a lot for their eating behavior?’) to identify social influence agents that exert power on health behaviors. In addition, there are different ways of analyzing the position or ‘centrality’ of individuals within networks. For example, a person receiving the most nominations has a high ‘in-degree’ centrality. Although this method is commonly used, it might be that ‘closeness’ centrality is a more effective measure because it describes how long it takes to spread information from that person to others (i.e., it takes into account the shortest paths a health message has to travel to reach the entire network). Another option would be to focus on ‘betweenness’ centrality, which indicates whether a person is important for linking different individuals or subgroups/clusters of individuals [20, 21]. To determine which individuals are the ideal social influence agents for the targeted behaviors of the *MyMovez* project, the selection process of social influence agents is investigated by focusing on different network centrality measures and by examining traditional (advice, leadership, friendship, etc.) and specific nomination items with regard to communication strategies, energy intake and expenditure.

It is also essential to identify which factors determine a person’s network position and which determinants mediate or predict engagement in energy behaviors. For example, research has shown an important role of ingratiation with regard to social normative and modeling behavior [13, 14]. That is, people try to influence each other to become part of a group and to be liked by conforming to social norms or modeling behavior. However, not all individuals are equally susceptible in doing so. Therefore, psychological determinants related to affiliation purposes and uncertainty reduction are collected such as need to belong, fear of negative evaluation, self-esteem, and athletic competence [13]. Nevertheless, research has focused mainly on characteristics of the followers and less on those of the social influence agents. There are limited indications that socially warm persons exert larger social influence than persons acting cold and that social influence agents have leadership status [8, 22]. To examine the characteristics of the influencers, Phase I also examines factors such as opinion leadership, public individuation, pro-social behavior, and participants are asked to profile themselves by choosing seven personality traits that describe themselves best [8, 21, 23, 24]. In addition, information about the social and physical environment is gathered that co-determine youth’s network positions and health behavior such as socio-economic status, social support, accessibility and availability of foods, and PA facilities [25].

The *MyMovez* project also uses a combination of renowned approaches and theories that predict behavioral change and provide insights in the performance of health behaviors and dissemination in social networks. For example, the constructs of the widely used Theory of Planned Behavior (TPB) are examined. The TPB has the general idea that when people have a positive attitude toward behavior and their social environment is supportive while they also feel they are capable and in control of their behavior, their intention to perform the behavior will be strong [26]. Importantly, people often fail to carry out healthy behaviors despite their positive intentions to eat healthy and engage in physical activity [27]. Therefore, additional constructs contributing to young people actually carrying out targeted behaviors (e.g., motivation [28, 29], self-persuasion [30], injunctive and descriptive norms [31], social modeling and impression management [32, 33]) stemming from the Diffusion of Innovation Theory, Social Normative approach [34], Self-Determination Theory [28] and the Fogg Behavior Model [29]), are examined to deepen the understanding of youth’s energy intake and expenditure. These insights from Phase I will contribute to develop an effective social network campaign for Phase II.

### Part B: Development of the *Wearable Lab*

A practical and modern way to conduct large scale research among youth is via smartphones, because it is an efficient, low-cost and less time-consuming method compared to traditional paper-and-pencil questionnaires. In addition, objective measures of health and real-time data can be collected. Furthermore, the social context can be incorporated by facilitating communications via social network functionalities. Part B of this comprehensive research project develops the *Wearable Lab*, existing of a highly innovative smartphone-based research application (*MyMovez* app) connected to an activity tracker. The research technology enables examining youth’s social network structures together with energy balance-related behaviors, media consumption, socialization processes and psychosocial determinants of behavior. The smartphone and activity tracker jointly collect self-reported and objective data on daily randomized and fixed time points. Each participant receives the *Wearable Lab* during each data wave for a period of 7 consecutive calendar days including the weekend.

The *Wearable Lab* is developed in an ongoing iterative process by means of focus group sessions as well as evaluations via the *MyMovez* app during the data collection waves. In 2015, six focus group sessions were conducted at primary (*N* = 30; 10-11 y/o) and secondary schools (*N* = 27; 12-13 y/o); with groups of boys or girls only and one mixed group at each school. Discussions were videotaped and led by trained researchers, and involved topics such as likes and dislikes of health, research apps and wearables, the design and attractive functionalities or features of the app, wearables and behavior tracking, and the project name. Outcomes contributed to choosing the name ‘*MyMovez*’ for the research project and co-creating the design and features of the app such as an avatar, game and social media platform called *Social Buzz*. At the end of 2016, six focus groups were conducted at two secondary schools ((*N* = 14; 12-14 y/o and *N* = 28; 12-14 y/o, respectively); with groups of boys or girls only and one mixed group at each school) to gather information about their social media use and to test the implementation of the social network functionality *Social Buzz*. All together, these focus group studies provided useful insights on youth’s attitudes concerning the design of the research application and practical information about the use of the *Wearable Lab*.

During Phase I, novel procedures and approaches to process and analyze data are explored. In addition, collaborations are set up, for example, to process photo data, analyze youth’s physical environment by connecting real-time data with Geographic Information System (GIS) measures, and analyze chat conversations from the *Social Buzz*. This also requires up-to-date safety and data management procedures according to legal and ethical standards. In sum, the *Wearable Lab* provides an innovative research methodology to collect data that increases the feasibility to implement and evaluate health campaigns.

## Methods/design

### Ethics statement

The research project is conducted according to the guidelines described in the Declaration of Helsinki and all procedures were approved by the ethical committee of the European Research Council (617253) and the Faculty Social Sciences, Radboud University, Nijmegen in the Netherlands (ECSW2014-100614-222).

### Design

The *MyMovez* project consists of a cross-sequential design, following two cohorts (8-12 and 12-15 y/o) over 3 years. Five waves of data are collected during the months February/March 2016 (w1), April/May 2016 (w2), June/July 2016 (w3), February/March 2017 (w4) and February/March 2018 (w5). Outcome measures involve social network characteristics, social media use, physical activity, beverage and snack consumption.

A power analysis was performed based on results from a previous cross-sectional longitudinal study that investigated energy balance behaviors among Dutch youth [35]. It was assumed that obesity inducing risk behaviors are present among approximately 40% of adolescents, and with a statistical significance level of .05 and 80% power, a sample size of 600 participants would be sufficient for the cross-sectional and longitudinal study. This sample size was also deemed appropriate for social network analyses, which require approximately 20-25 complete classroom networks. Assuming that Dutch school classes exist of an average of 25 kids, this would produce a sample between 500 and 625 students. Taking into account a conservative 40% attrition rate [36] we aimed to include 850 participants and oversampled the high school population because this age group is more likely to change schools or classes or drop out while followed during a period of three years. In the end, this led to recruitment of a sample of 953 participants.

### Recruitment of schools and participants

**Fig. 1:**
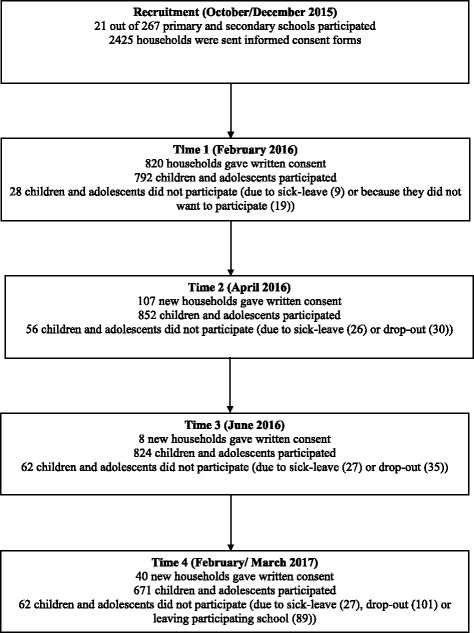
Flow diagram of the MyMovez recruitment procedure

All (sub)urban schools following a regular education program were eligible for participation ranging from primary school level, and lower vocational training to secondary school. All schools located near the Nijmegen area were invited to participate in the *MyMovez* project. Further, Dutch Public Health Services (GGD) of each province were contacted to promote the *MyMovez* project at their regions. In addition, schools were invited to participate via personal contacts of students and researchers. This random procedure resulted in the participation of 21 (sub)urban schools throughout the Netherlands for Phase I of the *MyMovez* project. See Fig. 1 for a flow diagram of the recruitment procedure.

After obtaining (written) consent of the school’s principals, parents/legal guardians received an information letter and/or e-mail via the school announcing the project’s goals, planning, execution and safety procedures. Parents/legal guardians could enlist their child to the *MyMovez* study by giving consent on either a paper-and-pencil or digital form on the *MyMovez* website (www.mymovez.nl). The *MyMovez* website provides easily accessible information and updates about the project, team and progress (e.g., by a video illustrating how a day looks like for a participant, Q&A page, photos, and infographics). Researchers also visited schools to explain and promote the project in school classes and to hold information meetings for teachers, student mentors, and parent advisory boards.

Despite the schools’ and researchers’ efforts to promote the *MyMovez* project, parents/legal guardians did not always take notice of the research (e.g., the response rate at some schools was < 5%), resulting in disappointed pupils who wanted to participate on the day the research materials were handed out to their classmates. These pupils were given the opportunity to contact their parents/legal guardians to give their consent via the website or hand in a written consent form after the school break. In addition, pupils had the opportunity to participate during each of the following data collection waves, which has thus far resulted in different sample sizes for each data wave (in combination with drop out cases): *n* = 792 in Wave 1; *n* = 852 in Wave 2; *n* = 824 in Wave 3 and *n* = 671 in Wave 4. It is expected that the participation rates for Wave 5 (February 2018) will differ as well due to attrition and new participations.

### Wearable lab

In this comprehensive project, the innovative research tool *Wearable Lab* is developed to collect a unique longitudinal dataset according to the highest standards in ethical and legal safety regulations. The smartphone with the *MyMovez* research app is connected to an activity tracker that both generate different types of data that are transferred wirelessly to a secured data server. Data types are self-reported and peer-reported (i.e., short surveys and sociometric queries, respectively) and experience sampling data, *Social Network Beacon* data, real-time physical activity data (i.e., steps and cycling) and location information, photos and chat conversation data on individual and class level by the social media platform *Social Buzz* (added to the *MyMovez* research app since January 2017). These data are used to examine youth’s media and social network exposure, socialization experiences, psychosocial determinants of behavior, and energy balance-related behaviors.

Social network information is gathered by asking youth to nominate peers with various characteristics (i.e., who is a leader?) as well as by recording chat connections and reports between participants on the social media feature *Social Buzz* in the *MyMovez* research app. In addition, social network information is also collected using the *Social Beacon Network*, in which the smartphones detect and register the research phones of interaction partners during multiple time points a day via Bluetooth. These data collection methods collectively provide insights into micro-processes (with multiple measurement points during a day) as well as macro-processes (multiple assessments across a school year). Furthermore, these methods provide information about youths’ perceived networks (i.e., sociometric items) as well as more objective assessments of networks based on physical proximity (i.e., the *Social Beacon Network)*.

Furthermore, the *Wearable Lab* allows for the collection of other novel types of data such as photo queries and global positioning system (GPS) data. For example, photo data are coded and used for machine learning purposes (e.g., coding of food or media use pictures) contributing to the examination of youth’s diets, social media behavior and other daily activities [37]. The GPS data provides information about cycling^1^ (the activity tracker only counts the amount of steps and the intensity) and the physical environment, by linking these to Geographic Information System (GIS) data in which youth are exposed to food and PA related facilities [38]. All together, the *Wearable Lab* allows for the collection of a comprehensive set of data that provides the opportunity to address all of the project’s research goals.

Participants receive surveys, photo questions or notifications on daily randomized or fixed time points. In addition, the researchers can personalize for each school class the time schedule in which participants are allowed to receive questions and notifications (i.e., before and after school and during school breaks only). Regularly used answering categories are already available in the backend of the app which saves researchers time in programming the surveys. In addition, it is possible to include follow-up or feedback loops based on answers to closed questions or PA measured by the activity tracker, send jokes/memes, riddles, and *MyMovez* news flashes. To keep the participants engaged, the app also includes two games (Zoko and Snake; which are allowed to play 5 min./hour) and participants can create and adjust their own avatar (e.g., face characteristics, hairstyle and -color, and additional attributes such as animals, music instruments, fruits, or sport gear). Participants also have the opportunity to chat with their classmates or with a researcher to ask research related questions, report misconduct, or ask for help.

### Procedure

Most schools were visited in advance to promote the research project among the participants before the project started. On the starting day of the initial data collection, trained researchers handed out the research materials (i.e., smartphone and activity-tracking bracelet) and instructed the participants in class or in small groups of approximately 5-8 school children about the use of the *Wearable Lab*. Specifically, it was explained how to turn the phone on and off, how to use the social chat and avatar, how to check whether the activity tracker is connected to the smartphone, how to check whether the batteries are full, and what to do when the phone displays an error. Participants were also told that they should wear the activity bracelet for the entire week and that the activity bracelet is water resistant. Participants were also shown a short movie clip about how a research day with the *Wearable Lab* looked like and they were handed a code of conduct showing how to behave in the *Social Buzz* social media platform. Next, participants signed an assent form and an additional agreement in which they promised to handle the research equipment with care during the 7-day period they were borrowing the research materials. Participants were informed that they could withdraw from the research at any moment, and that their data can be deleted if they or their parents want to. After the instructions they were provided with the opportunity to ask questions and could return to their classrooms. Each year (during Wave 2, 4, and 5), participants’ weight and height is measured individually in a separate room by a trained researcher. Depending on the school schedule, this is done after the instruction or participants are called a second time from their classroom.

On the starting day of each measurement period, participants receive questions after their school day ended. During the following days, they all receive questionnaires at random time points during the day, but never during school hours or at night between 7:30 PM and 7:00 AM. At the end of the measurement period, reminders are sent to the participants when and where they need to hand in the research materials at their schools. Researchers collect the research materials at the schools.

### Measurements

Data collection involves explicit self- and peer-reported survey measures (short digital questionnaires administered via the smartphones), objective measures (photos, real-time data, activity tracking, cycling, *Social Beacon Network* and *Social Buzz* data), and researcher-assessed measures (participants’ height and weight). Most survey measurements used in the *MyMovez* project are existing validated questionnaires, translated into Dutch. Some (parts of) questionnaires are adapted from validated questionnaires and tailored to identify specific behaviors. In cases in which no validated measures are available, new items or scales are developed for the *MyMovez* project. All measurements used in the *MyMovez* project described in the following paragraphs. The survey instruments are listed in Table 1 and described into detail in Additional file 1.

**Table 1:** Overview of survey measures MyMovez project – Phase I

	Content/captures	Instrument(s)	Reference(s)
Individual measures			
	Self-esteem	Rosenberg Self-esteem scale	[49]
	Body esteem	Figure Rating Scale	[50, 51]
	Need to belong	Need to belong Scale	[52]
	Fear of Negative Evaluation	Brief Fear of Negative Evaluation Scale	[53]
	Happiness	The Faces Scale	[54]
	Pro-social behavior	Strengths and Difficulties Questionnaire	[55]
	Public individuation	Public individuation	[56]
	Self-profiling of personality characteristics	Descriptive	[57]
Behavioral and motivational constructs of energy intake and expenditure			
	Theory of Planned Behavior (attitude, self-efficacy, descriptive and injunctive norms, and intentions)	Theory of Planned Behavior constructs	[26, 58, 59]
	Motivation	Adapted measures from the Health Care SDT Packet Perceived competence scale	[60]
	Fogg Behavior Model (determinants motivation and ability with antecedents pleasure/pain, hope/fear, and social acceptance/rejection, time, money, physical effort, brain cycles, social deviance, and non-routine)	- Fogg Behavior Model - Theory of Planned Behavior constructs - Adapted measures from the Physical Activity Enjoyment Scale PACES - Adapted measures from the Perceived Benefits of Physical Activity Scale - Adapted measures from the Self-Report-Habit-index Scale (SRHI)	[29] [58, 59] [61] [62] [63]
	Opinion Leadership	- King and Summers Opinion Leadership scale - Opinion Leadership scale	[23] [24]
	Perceived social support	Social support scale	[64]
Energy intake related measures			
	Consumption behavior (hunger, thirst, including specific Dutch snack and drink items)	- Food Frequency Questionnaire (FFQ) based on the Dutch EPIC Frequency Questionnaire	[65] [12]
	Role models or ‘prototypes’ of consumption behavior	Prototype-Willingness Model	[66]
	Self-regulatory resources (internal and external attribution)	Nutrition Locus of Control	[67]
	Dieting behavior	Descriptive	
	Physical environment diet (availability and accessibility)	- Questionnaire to assess determinants related to fruit and vegetable intakes in children - Child-reported family and peer influences questionnaire	[68, 69]
	Parental role modeling	adapted from the Home Environment Scale	[70]
	Occurrence unhealthy eating behaviors	Descriptive / filler items	
Energy expenditure related measures			
	Athletic competence	Perceived Competence Scale for Children	[71, 72]
	Barriers to Physical Activity (body-related, resources, social, fitness, and inconvenience barriers)	Barriers to PA scale	[73]
	Daily activities and sports participation (which sports they play and like)	Descriptive / filler items	
	Environmental factors	Descriptive	[74]
	Habitual physical activity	- Activity Questionnaire for Adolescents and Adults - Questionnaire to Assess Health-enhancing physical activity - ‘Day in the life’ Questionnaire	[75] [76–78] [79]
	Social norms and perceived physical activity	Adapted from TPB constructs	[58, 59]
	Injury check	Descriptive	
	Motives for being physically active	The Self-presentation Motives for Physical Activity Questionnaire	[33]
(Social) Media consumption			
	Television (TV) exposure (duration of exposure, type of TV stations, genre TV programs)	Descriptive	[80] [81]
	Internet and social media exposure (duration of exposure, type of social media platforms)	Descriptive	[80] [81]
	Video blog (Vlog) exposure (duration of exposure, names of Vloggers, type of Vlogs)	Descriptive	
	Gaming behavior (duration, type and genre of games, which devices used, most liked)	Descriptive	
	House rules on media use (screen-based activities)	Activity Support Scale for Multiple Groups	[82]
	Mealtime media use	Descriptive / filler items	[83]
	Use of health apps and Wearables (most liked, app effectiveness)	Descriptive / filler items	
Research and app evaluation			
	Evaluation research and app (liking en enjoyment research and app, games, avatar, jokes and memes, and *Social Buzz*)	Descriptive / filler items	
Filler items			
	Naming favorite foods, music, brands, animals, movies, celebrities, sports and sports heroes, daily planning, etc.	Descriptive / filler items	

Outcome variables (e.g., social network characteristics, physical activity, drink and snack consumption), as well as TPB and motivational measures, are measured at each data wave assessment. Other measures are divided over the different data waves. A detailed scheme can be provided upon request to the corresponding author. Participants’ name, birth date, gender and postal code were provided by the school^2^ to create an ID number in the back-end of the *MyMovez* research application.

#### I. Survey measures

##### Demographics

Self-reported demographics are assessed in a survey during the start of the project (and for every new participant that enrolled in later waves), consisting of participant’s age, sex, grade level, nationality, caregivers’ nationalities, and whether they have older and younger, male or female siblings.

##### Body mass index (BMI)

Participants’ weight and height are measured individually by a trained research assistant according to standard procedures (without shoes but fully clothed) in wave 2, 4, and 5. Height is measured to the nearest 0.1 cm using a stadiometer and weight to the nearest 0.1 kg using a digital scale. Participant’s Body Mass Index (BMI) is calculated using the standard formula weight [kg]/height2 [m]. The BMI-scores are standardized by using the LMS-method, which accounts for variations in growth curves of children and adolescents of different ages and gender for different countries. BMI (z-score) cutoff points were used representing the current z-BMI standards for Dutch children and adolescents [39]. Self-reported measures about height en weight are assessed when participants entered the project after Wave 2 (when weight and height were measured by the researchers).

##### Socioeconomic status

The socioeconomic status of the participant’s family is measured with the second version of the Family Affluence Scale (FAS II) [40]. This scale consists of six items concerning the number of computers, laptops or tablets, cars and bathrooms in the household, and a question about the number of holidays the family could afford each year. In addition, it is asked whether the participants have their own bedroom, and whether there is a dishwasher in the household. Answering categories about the bedroom and dishwasher are dichotomous. Other answering categories ranged from ‘*None*’ (0) to either ‘*Two or more*’ (2) or ‘*Three or more*’ (3). The sum score of the scale (ranging between 0 and 13) indicates a higher socioeconomic status. In addition, the participant’s postal codes provide information about their socioeconomic status. The neighborhoods in which participants live are classified on socioeconomic status by Statistics Netherlands.

#### II. Sociometry and social beacon network

##### Sociometric questions

To map social network structures and identify social influence agents for the health behaviors, participants are asked to complete a total of 16 peer nomination items during the course of the research week (approximately 3 questions per day). The class name list provided by the schools are uploaded into the backend of the *MyMovez* app, so participants can scroll down the list and tick off names of peers who are in the same grade regardless of their participation in the *MyMovez* project.^3^ Participants could nominate an unlimited number of peers for each item, but could not nominate themselves. Participants are required to nominate at least one peer for each item.

The general network questions are based on previous research [9, 12, 19]: ‘*Who are your friends?*’, ‘*Who do you ask for advice? With this we mean children from you class or school whose opinion you value.*’, ‘*Who are leaders or take the position of leader in a group?*’, ‘*Who do you respect? With this we mean children you admire, because they, for example, are good at something.*’, ‘*With whom do you spend time?*’ In addition, questions are added to detect innovators (‘*Who often has new gadgets and clothes?*’) and ask about modeling behavior (‘*Whom do others want to look like?*’).

Additionally, participants are asked to nominate peers for each of the specific health behaviors (dietary intake, physical activity, and media use) to detect innovators, role models, impression managers, and messengers. For example, ‘*With whom do you talk about what you eat or drink?*’ (dietary communication), ‘*Of whom do you think it is important that they see you as someone who is physically active?*’ (PA impression management), and ‘*Who eats or drinks products that you would like to eat or drink?*’ (dietary modeling).

In addition to the sociometric questions, participants are also asked from which source they like to receive advice about eating healthy snacks, physical activity and media use, and would follow advice from, with several reference groups as response options (e.g., peers, older adolescents, celebrities, vloggers, parents, experts, teachers).

##### Social beacon network

Social networks are also assessed with Bluetooth technology to detect proximal peers via smartphones. The *Social Beacon Network* is an innovative unobtrusive way to measure physical distances between all participants. The research smartphones scan and detect other participants’ phones that are within Bluetooth range (approximately 10 m). When participants are within close proximity for more than half an hour, two questions are triggered to check whether participants are actually spending time together and to avoid measurement error caused by participants passing by accidentally while the phone was scanning the proximity (‘*Are you spending time with someone?*’ If so, ‘*With whom are you spending time?’*). In addition, they are asked to indicate what they are doing in an open-ended format. The Social Beacon Network provides one way of validating the sociometric questions and the social networks created from these items.

In conclusion, the *Social Beacon Network* can provide insights into the relative frequency of interactions between individuals (with multiple measurement points) during the day, which complement the sociometric items, which are only assessed once per data collection wave. This unobtrusive research method requires new data management strategies that are being developed during this project.

#### III. Activity tracking bracelet

Participant’s activity tracking bracelet *Fitbit Flex* (i.e., accelerometer) measures physical activity as the number of steps in continuous time (i.e., minute to minute). The bracelet calculates active minutes using metabolic equivalents (METs). METs are widely used as indicators for exercise intensity measuring in a comparable way among persons of different weights. The intensity of the activity is categorized in sedentary activity, light intensity activity, moderate intensity activity, and vigorous intensity activity, in line with the Center for Disease Control’s (CDC) recommendations [41]. The number of steps each day, as well as the number of minutes in each of the PA categories recorded on each Fitbit is automatically transferred to the *MyMovez* app. The first and the last day of the measurement period are excluded because the participants do not wear the bracelet the entire day (i.e., the research materials were given to and received from participants during school hours).

#### IV. Real-time data

The Netherlands is famous for its cycling culture and almost every household owns at least one bicycle [42]. Because the wearable accelerometer is worn at the wrist, the device does not detect physical activity while riding a bicycle. Therefore, to have a complete assessment of PA for Dutch youth, cycling data is obtained to complement the activity detected by the accelerometer. On the basis of the velocity patterns measured by the accelerometer in the smartphone, the software estimates the location of smartphone. Based on the GPS locations of the beginning and the end of the cycling trip, the average speed and distance is calculated.

The GPS data also provide information about the physical environment of the participants to investigate the proximity of facilities conducive to snack consumption and physical activity (e.g., snack bars, swimming pools, parks, etc.).

#### V. Photos

Participants receive two photo questions at random time points during the day in each data wave from day 2 to 5. They are asked to make a picture of what they are doing at that moment [43]. When the photo is taken, a question is triggered so that the participants can categorize the photo into ‘reading or doing homework’, ‘watching TV/tablet/computer’, ‘gaming’, ‘having a snack, drink or meal’ or ‘other.’ Depending on their answer, they are directed to follow-up questions to explain their activity in further detail (e.g., which game they are playing) and describe what is in the picture they photographed. In addition, the photos are coded for detailed content. Before the coding of the photo data by independent coders, a trained researcher of the *MyMovez* team inspected all the photos to ensure that no identifiable or sensitive information is being shared. A codebook is available on request.

#### VI. Social buzz chats

Starting in January 2017 (Wave 4), participants are able to chat with each other in the social media platform *Social Buz*z, which is a safe chat environment within the *MyMovez* app. *Social Buzz* involves three chat levels, with separate time lines. Participants are able to talk with their classmates 1-on-1 (individual time line in the *MyBuzz*), with their classmates in a group chat (class-level time line in the *ClassBuzz*), and with the *MyMovez* research team (the *MyMovez* time line). Besides chat conversations, participants have the opportunity to like, share and respond to messages or images sent by their peers or by the *MyMovez* team. Images can be sent from picture folders that are composed by the *MyMovez* team. By January 2018, participants will also be able to like, share and comment on short video clips. For privacy reasons, it is not possible for participants to send pictures, images, or videos they make or create themselves. The *MyMovez* research team has the opportunity to send messages to the participants as well (individual level or class level).

Within the *Social Buzz* environment, we integrated the option for participants to flag inappropriate behavior. When participants use the flag button, for example, when they see a message that they do not think is appropriate, researchers from the *MyMovez* team receive a notification so that they can solve the situation immediately. *MyMovez* team members have the possibility to remove specific messages that are flagged, block specific users, or shut down the entire *Social Buzz* option for a class. The *Social Buzz* is also very useful for *MyMovez* researchers when they want to communicate with the participants, for example to remind them to hand in their *Wearable Lab*.

The purpose of the *Social Buzz* is two-fold: On the one hand, it reinforces participant’s engagement in the project, on the other hand it provides the opportunity to investigate social media use and social network relations (i.e., who are connected to each other). In Phase II, the *Social Buzz* will be used to implement and test intervention messages which will provide interesting insights in how intervention messages disseminate through social networks and which strategies are used to stimulate or discourage health behaviors in social networks. The *Social Buzz* data can be exported (from January 2018) into four separate data log files in which 1) group and individual messages are listed, 2) all given likes are listed which can be traced back to the source, 3) media exposure are listed (e.g., which video and duration of watching), and 4) a summary file is listed composed of several variables from the other 3 files. These log files are available on request.

#### Strategy of analysis

A variety of statistical techniques are used to address the project aims. In order to identify the most powerful influence agents traditional centrality measures (e.g., indegree centrality) are utilized, as well as more advanced techniques capable of identifying influence agents, such as “greedy” search algorithms available in the KeyPlayer package in R (R Core Team, 2015) [44]. To examine similarity and social influence of health behaviors in youth’s social networks, dyadic modeling techniques, such as actor-partner interdependence models [45] and stochastic actor-oriented models of network and behavioral co-evolution are used [46]. These modeling techniques are specifically designed to account for the known interdependencies in relational data. The stochastic actor-oriented models are particularly useful because of their capability to initially assess similarity in health behavior among friends, and also to disentangle processes associated with peer selection (i.e., creating or dissolving relationships on the basis of health behaviors) from peer influence (i.e., youth adapting the health behaviors of their peers).

In order to investigate which factors determine youths’ network positions as well as their energy intake and expenditure, multiple linear regression analyses, multilevel regression analyses, structural equation models (SEM) and mixed effect models are used to fit the observed data to theoretical constructs, and test the predicted relationships. Due to the innovative features of this project, some of the generated data require exploratory data analysis and sometimes without golden standards to refer to (e.g., *Social Network Beacon* data). Analyses are conducted with various statistical programs, including SPSS (version 24; IBM Corp., 2016), Mplus (7) [47], and R (R Core Team, 2015).

## Discussion

The *MyMovez* project-Phase I is a comprehensive multidisciplinary research project that extends existing knowledge by investigating the interplay between social network influences in relation to individual and environmental factors for youth’s energy-balance behaviors in a contemporary social media landscape. Moreover, the cross-sequential design allows for the assessment of changes within participants over time as well as differences between the two age cohorts. This pioneering project explores new standards for social network research, processing data and eventually the implementation of media health campaigns (Phase II of the project). The systematic way in which youth’s social networks are investigated by focusing on different social network aspects (i.e., types of questions and centrality measures for each of the health behaviors) in conjunction with individual and environmental (physical, social, and media) factors make this research project unique. The *MyMovez* project contributes to theory development by bringing together several approaches and theories of media and marketing effects, behavioral change, socialization, and network dynamics [8, 26, 32, 34]. In addition, this project develops the *Wearable Lab* by which the findings do not rely solely on self-reported measures but also on objective and real-time measures. The Wearable Lab supports keeping youth motivated and engaged to participate in the *MyMovez* project by adding a modern and fun factor.

There are some limitations to be discussed concerning Phase I of this project. For example, participation in the *MyMovez* project requires obtaining active consent from school principals, parents/legal guardians, and participants. Although the enrollment procedure is made as easy as possible (e.g., through online registration via a link or QR code as well as paper-and-pencil forms), we experience that parents/legal guardians are occupied with their daily routines and often forget to enroll their child. In addition, some parents/legal guardians may find the project too engaging or even intrusive. As a result, participation rates differ within classes. Participation rates lower than 60% per class affect social network analysis, leading to less power and validity of the results [48]. In addition, it is quite common that in longitudinal research, participation rates vary across data waves due to attrition and in our case because participants can enter each wave or do not finish the 7-day research period. Missing data can cause restrictions on the validity of the research findings; however, having obtained some participant data enables imputing missing data to solve this limitation. Another challenge in this exploratory project is the innovativeness of the methods used. For example, the *Social Beacon Network*, activity tracking among young people, using real-time data and social chats and photos require unconventional data analysis strategies and management protocols. The *MyMovez* project team takes great care of keeping up to date with current developments and is open for collaborations with other researchers.

In sum, the *MyMovez* project contains a rich data set that allows to investigate how various social influences co-determine youth’s energy intake and expenditure in combination with individual, psychosocial and environmental factors. The technological features of the *Wearable Lab* advances research due to the development of novel analytical and research strategies and the added fun-factor that contributes to the motivation of the participants. The project is theoretically and methodologically pioneering and will produce a unique and feasible method for improving large-scale media health campaigns. The *MyMovez* project-Phase I will give new theoretical and practical directions to scientific settings and intervention studies using social network interventions.

## Additional file

Additional file 1: Detailed description of survey measures MyMovez project – Phase I. Detailed description of all measures administered in the MyMovez project – Phase I by the *Wearable Lab*: individual measures, personality traits, behavioral and motivational constructs of energy intake and expenditure, energy intake and expenditure related measures, (social) media consumption, research and app evaluations and filler items. (PDF 279 kb)

## Abbreviations

GGD: Dutch Public Health Services
GIS: Geographic Information System
GPS: Global Positioning System
PA: Physical Activity
Q&A: Questions and Answers
TPB: Theory of Planned Behavior
VAS: Visual Analogue Scale

## Footnotes

1 Cycling data is an important aspect of youth’s PA in the Netherlands

2 Some schools only provided just participants’ names. Additional info was then gathered via self-report survey measures.

3 The *MyMovez* app also provides the possibility to tick off names of other class lists within the same school year; however, this data is not suitable for further analysis because the participation rate is too low for creating a social network on school level.
