# Supplementary material for: Youth’s social network structures and peer influences: study protocol MyMovez project – Phase I
Source: BMC Public Health. 2018 Apr 16;18:504. doi: 10.1186/s12889-018-5353-5 (PMC5902932; doi:10.1186/s12889-018-5353-5)
Supplement: Supplementary file 1 — Detailed description of survey measures MyMovez project – Phase I. Detailed description of all measures administered in the MyMovez project – Phase I by the Wearable Lab: individual measures, personality traits, behavioral and motivational constructs of energy intake and expenditure, energy intake and expenditure related measures, (social) media consumption, research and app evaluations and filler items. (PDF 279 kb) [file 12889_2018_5353_MOESM1_ESM.pdf]

Individual measures and personality traits

Self-esteem. Self-esteem is measured by the Rosenberg self-esteem scale which is a widely used 10-item questionnaire measuring both positive and negative thoughts about oneself [49]. Items are framed as '*How often…*' participants felt worthy or not about themselves (e.g., '*How often are you feeling satisfied about yourself?*') with answering categories ranging from '*Never*' (1) – '*Always*' (6).

Body esteem. Body esteem is measured by the Figure Rating Scale (FRS) consisting of 9 silhouette drawings ranging from very thin (1) to obese (9) [50]. Participants indicate which silhouette number corresponds with their perceived current body figure and the number that they regard as their ideal body figure. The discrepancy between the ideal figure and perceived current figure represents their body esteem. A score below zero indicates a lower body esteem (i.e., a larger body dissatisfaction) and a score of '0' indicates that participants are satisfied with their appearance. A score above '0' also indicates body dissatisfaction (for boys, this usually means that they want to become more muscular) [51]. This means that analyses has to deal with the nonlinearity of the scale. For example, by coding all positive values as their negative equivalent, the scale gives insights into body dissatisfaction in general but no distinction can be made between individuals who want to lose or gain weight. Another possibility is to perform analyses on a subsample (e.g., individuals who want to lose weight vs. those who are satisfied). Both of these strategies are used within the *MyMovez* project.

Need to belong (LTB). Need to belong is measured with 10 statements assessing the desire to develop or maintain interpersonal bonds (e.g., '*I need to feel that there are people I can turn to in times of need*' or 'I *try hard not to do things that will make other people avoid or reject me*') [52] with answering possibilities ranging from '*I completely disagree*' (1) – '*I completely agree*' (6).

Brief Fear of Negative Evaluation Scale (BFNE). The fear and distress of being evaluated unfavorably by others in a social situation is measured by the 12-item BFNE [53]. Items are prefaced with '*How often…*' participants feel distress or social anxiety with answering categories ranging from '*Never*' (1) – '*Always*' (6) (e.g., '*I am frequently afraid of other people noticing my shortcomings*' or '*I often worry that I will say or do the wrong things*').

Happiness. Participants indicate their overall happiness by rating how happy they are '*most of the time*' on The Faces Scale [54] on the first day of each assessment. Participants choose between 7 faces gradually transforming from very happy to neutral to very sad. On the succeeding research days, the participants receive randomly a question about how happy they

are at that moment. They indicate their happiness on a Visual Analogue Scale (VAS) by a slider ranging from ‘*Very unhappy*’ (0) to ‘*Very happy*’ (100).

Pro-social behavior. Pro-social behavior is administered by a five items subscale from the self-report Strengths and Difficulties Questionnaire (SDQ) (e.g., ‘*I try to be nice to other people*’, ‘*I usually share with others (food, games, pens, etc.)*’or ‘*I am kind to younger children*’) [55]. Each item is answered on a 6-point scale ranging from ‘*I completely disagree*' (1) – '*I completely agree*' (6).

Public individuation. Public individuation [56] is measured with 12 items on a 6-point-scale ranging from ‘*No, certainly do not*’ (1) – ‘*Yes, certainly do*’ (6). The items measure whether participants find it easy to perform in public such as giving a lecture in front of many people, raising their hand in class, or taking the lead in a group of unfamiliar peers.

Self-profiling of personality characteristics. Participants are asked to describe themselves by choosing 5 characteristics from a list of 59 characteristics in total. The list contains positive, neutral and negative items such as being curious, role-model, smart, fast, clumsy, helpful, sporty, popular, sweet, quiet, creative, aggressive, cool, stressed, brave, adventurous, shy, etc. In addition, participants are asked to indicate whether they think their personality is linked to their performance and decision making by using their head or following their heart [57].

Behavioral and motivational constructs of energy intake and expenditure

Theory of Planned Behavior (TPB). The TPB constructs (attitude, self-efficacy, descriptive and injunctive norms, and intentions) are assessed separately for energy intake and expenditure behaviors at each wave [26,58, 59]. Attitude is assessed by two items about enjoyment and liking (e.g., ‘*I find eating fruit and vegetables as a snack…*’ followed a 4-point scale ranging from ‘*Very unpleasant*’ (1) – ‘*Very pleasant*’ (4)). Self-efficacy (i.e., perceived behavioral control) describes to what degree participants think they succeed in performing the behavior and how easy or difficult the behavior is to perform, assessed with two items on a 6-point scale (e.g., ‘*Do you think you will succeed in exercising more?*’ and *‘Do you think it is easy for you to exercise?*’; ‘*No, certainly do not*’ (1) – ‘*Yes, certainly do*’ (6)). Norms are assessed separately for parents and friends by inquiring about injunctive (i.e., subjective) as well as descriptive norms on a 6-point scale ‘*No, certainly do not*’ (1) – ‘*Yes, certainly do*’ (6) and ‘*Never*’ (1) – ‘*Always*’ (6), respectively). Injunctive norms are assessed with two items: ‘*Do you experience that your parents/caretakers think you should eat fruit and vegetables as a snack?*’ and ‘*How often do your friends encourage you to eat vegetables or fruit as a*

snack?' The descriptive norm (i.e., modeling behavior) is assessed with one item asking the frequency that caregivers or friends perform the behavior ('*How often do your caregivers drink water?*')[59]. The intention to perform the behavior is assessed with one item asking how certain they are to perform the behavior ('*Do you intend to drink more water?*'; '*No, certainly do not*' (1) – '*Yes, certainly do*' (6)') [58].

Motivation. Several motivational measures are also assessed separately for energy intake and expenditure behaviors at each wave (questionnaires are adapted from the Health Care SDT Packet) [60]. Intrinsic motivation is measured with 4 items on a 6-point scale that describe the participant's liking, enjoyment, feeling pleasure and wanting to perform the behavior (e.g., '*Do you eat fruit and vegetables as a snack because you like it?*'; '*No, certainly do not*' (1) – '*Yes, certainly do*' (6)). Extrinsic motivation is also assessed with 4 items on a 6-point scale that describe participants' evaluations by others when they perform the behavior, such as feeling ashamed, thinking they are stupid, liking or excluding (e.g., '*Do you exercise because you are afraid to not fit in?*'; '*No, certainly do not*' (1) – '*Yes, certainly do*' (6)). Perceived Competence (based on the Perceived Competence Scale) [60] describes more general forms of motivation and the engagement in healthy activities. Competence is measured by 4 item statements (e.g., '*I am confident that I can eat and drink healthy*' and '*I will succeed in eating and drinking healthy'*; '*No, certainly do not*' (1) – '*Yes, certainly do*' (6)).

Fogg Behavior Model (FBM) [29]. FBM is measured by the main determinants motivation and ability (i.e., self-efficacy) and their antecedents for both consumption behavior and physical activity. Motivation is assessed with intrinsic and extrinsic motivation items (as described in the above). Ability is assessed with the self-efficacy items of the TPB (as described in the above).

According to the FBM, motivation is influenced by several anteceding variables [29]: pleasure/pain, hope/fear, and social acceptance/rejection. The pleasure/pain dimension is operationalized by adapting measures from the Physical Activity Enjoyment Scale PACES [61] (and also administered for snacking and drinking behavior). The adopted measures consist of 16 items (prefaced by the statement '*When I eat and drink healthy or exercise…*') that describe how participants feel when performing the behavior (e.g., liking, disliking, being bored, having fun, despising, giving energy, feeling happy/cheerful/successful, feeling frustrated) measured on a 6-point scale ranging from '*I completely disagree*' (1) – '*I completely agree*' (6). The dimension hope/fear is operationalized by adapting measures from the Perceived Benefits of Physical Activity scale [62]. These measures consist of 8 items

describing beneficial consequences of performing a behavior (prefaced by the statement '*When participants would eat and drink healthy/exercise regularly…*' and followed by, for example, '*…they would spend more time with friends'* or '*…improve their appearance or the way they feel about themselves*') with answers ranging from '*I completely disagree*' (1) – '*I completely agree*' (6). The acceptance/rejection dimension is assessed with 4 items measuring acceptance ('*approve*' and '*accept*') and rejection ('*think it is bad*' or '*disapprove*') (e.g., '*Do others approve when you eat or drink healthy/exercise?*' with answering categories ranging from '*No, certainly do not*' (1) – '*Yes, certainly do*' (6).

According to the FBM, ability is predicted by the antecedents [29]: time, money, physical effort, brain cycles, social deviance, and non-routine. The time, money (from both the participant and parent), and physical effort constructs are measured with single items on a 6-point scale (e.g., '*Do you think it takes you too much time to eat and drink healthy?*', '*Do you think it costs too much money to exercise?*' '*No, certainly do not*' (1) – '*Yes, certainly do*' (6), and '*How often does it take a lot of effort to eat and drink healthy?*' '*Never*' (1) – '*Always*' (6). Brain cycle is assessed by asking the extent to which a behavior is something to be reminded of (e.g., '*How often do you forget to eat and drink healthy?*') and is measured on a 6-point scale, ranging from '*Never*' (1) – '*Always*' (6). Social deviance is measured by use of the TPB descriptive norms for parents and friends. Non-routine is measured by use of Self-Report-Habit-index Scale (SRHI) [63]. The index consists of 12 items that assess the frequency of the behavior, to which extent it is automatic and how participants feel if or when they perform the behavior or not (e.g., '*Exercising is something I do regularly*' or '*Eating and drinking healthy is something that I have been doing all my life*'). The items were measured on a 6-point scale ranging from '*I completely disagree*' (1) – '*I completely agree*' (6).

Opinion Leadership. Different opinion leadership scales are administered about snacking behavior, PA and lifestyle in general. To measure participants' influence on others, the shortened King and Summers Opinion Leadership scale is adjusted to dietary behavior and physical activity [23]. Items are prefaced by '*How often…*' and ask about their communication with others about eating and drinking or being physically active (e.g., '*How often is your advice asked about eating and drinking?*' or '*How often do you tell your friends about being physically active?*'). Participants answer on a VAS ranging from '*Very little*' (0) – '*Very much*' (100). The lifestyle scale is adapted from the Opinion Leadership scale [24] and reduced to 5 statements focusing on a person's influence on other's general lifestyle (e.g., '*My opinion about a healthy lifestyle is important to others*' or '*I often influence how others*

think about their lifestyle') with answering categories ranging from '*I completely disagree*' (1) – '*I completely agree*' (6).

Social support. Perceived social support is assessed for friends and parents with a 9-item scale for healthy eating and PA. Participants rated how often friends or parents engaged in specific (un)healthy activities (e.g., '*How often…*' '*…do your friends offer to participate in physical activities with you?*' or '*…do you parents encourage you to eat healthy?*'), which is measured on a 6-point scale, ranging from '*Never*' (1) – '*Always*' (6) [64].

Energy intake related measures

Hunger and thirst. How hungry and thirsty participants are at the time they fill in the Food Frequency Questionnaire is measured by single items on a VAS scale, ranging from '*Not hungry/thirsty*' (0) – '*Very hungry/thirsty*'.

Snacking behavior. Snack intake is assessed with a Food Frequency Questionnaire (FFQ) including specific Dutch food items based on the Dutch EPIC Frequency Questionnaire [65]. Participants are asked every other day (2 weekdays and 1 weekend day) to recall how many snacks they ate the day before. In the drop down menu of the *MyMovez* app, they indicate how many pieces they ate ranging from '*0*' to '*6 or more*'. The questionnaire contains items about small cookies and biscuits, large cookies (e.g., muffins, piece of cake or pie), packaged cookies (e.g., Oreo, Sultana, Liga, Snelle Jelle), pieces of chocolate (e.g., pralines or bonbons), chocolate bars (e.g., Mars, Twix, Snickers), candy or liquorices, a handful of chips or salty snacks, salted nuts (e.g., salted mix nuts, peanuts), fruits, tablespoons of salad, vegetables (e.g., small tomatoes, pieces of cucumber), warm snacks (e.g., pizza slice, meat croquet, sausage roll, 'frikandel'), cube of cheese, slice of sausage, bowl of yogurt or cottage cheese and ice cream.

Beverage consumption. Participants are asked every other day (2 weekdays and 1 weekend day) to recall what they drank the day before. The questionnaire contains items about (sparkling) water, milk, unsweetened milk or yoghurt drinks (e.g., *Optimel*), sweetened milk or yoghurt drinks (e.g., *Fristi*, chocolate milk, *Milk&Fruit*), 100% fresh fruit or vegetable juice, juice (e.g., *Wicki, Dubbeldrank*), tea and coffee without sugar, tea and coffee with sugar, light sodas, regular carbonated sodas, lemonade based on syrup, energy drinks with caffeine (e.g., *Golden Power, Red Bull, Monster*), and energy drinks without caffeine. Response categories ranged from '*zero glasses per day*' (0) to '*seven or more glasses per day*' (7). Participants are instructed that one glass (or other drink packages such as cans) was approximately 200ml [12].

Prototype-Willingness Model. The prototype scale was constructed on the basis of previous studies concerning health related behaviors [66]. Social influence agents are regarded as 'prototypes' that are related to their peers' eating and drinking behavior [67]. To assess the degree to which participants compare consumption prototypes to their self-image (i.e., the more similar, the more likely they are to engage in the behavior associated with that prototype), participants first indicate which characteristics a peer who eats and drinks healthy possesses typically (e.g., someone who is smart, future oriented, satisfied, confident, active, well-groomed, etc.). Next, participants indicate how much they resemble that person (i.e., prototype). Finally, the willingness to eat or drink healthy is assessed by providing an example scenario in which they are offered an unhealthy food or drink by a friend. They have to indicate whether they would accept it, are able to refuse, and whether they would walk away from the offer. All answering categories are presented on a 6-item scale ranging from '*No, certainly do not*' (1) to '*Yes, certainly do*' (6).

Nutrition Locus of Control. The Nutrition Locus of Control scale measures the extent to which participants feel that they have control over their eating behavior or comply with external forces. Having lower self-regulatory resources may lead to more unhealthy food choices [67]. Items are measured on a 6-point scale ranging from '*I completely disagree*' (1) – '*I completely agree*' (6) on 8 concerned statements such as '*I believe that I have the responsibility to choose foods that are good for me*' or '*It would be simply a matter of luck if I happened to get healthy food every day*' (example items of internal and external attribution, respectively).

Dieting behavior. Participants are asked whether they adhere to a diet ('*Yes*' or '*No*'). If they give a positive answer, they are asked which diet in an open-ended question. They are also asked whether they fast due to Ramadan ('*Yes*' or '*No*').

Physical environment diet. The questionnaire items focus on availability and accessibility of vegetables and fruits at school and in the home environment (e.g., '*Is it possible to buy vegetables or fruit at school?*' or '*Are there vegetables and fruits available at home you like to eat?*') [68]. In addition, participants are asked whether parents/legal guardians would buy vegetables and fruit when participants ask for it, and whether caregivers involve them in meal preparation [68, 69]. The items are answered on a 6-point scale ranging from '*No, certainly do not*' (1) – '*Yes, certainly do*' (6) and '*Never*' (1) – '*Always*' (6), respectively. Additionally, questions are administered about whether there is a vegetable garden, snack bar and supermarket near their home environment or school ('*Yes*' or '*No*').

Parental role modeling. The measure of parental role modeling is adapted from the validated Home Environment Scale (HES) to assess parents' eating behavior and habits (i.e., role modeling) regarding healthy foods [70]. The original scale contains questions for parents about their child, but for the current research the questions are transformed to ask participants about their parents. Participants completed 6 items indicating the frequency of their parents engaging in certain behaviors, and are measured on a 6-point scale ranging from '*Never*' (1) – '*Always*' (6) (e.g., '*How often…*' '*…do your parents have dinner in front of the television*' or '*…eat unhealthy snacks such as chocolate, cookies, candy or chips*?').

Occurrence unhealthy eating behaviors. The time of the day that participants usually eat unhealthy snacks is assessed with choice options: '*during school breaks*,' or when they are '*with friends*,' '*coming home from school*,' '*watching TV*,' '*gaming*,' '*doing homework*,' '*behind the computer/laptop*,' or '*other*' (followed by an open-ended question). In addition, participants could choose from a list of 25 fruits and snack vegetable items (e.g., cucumber and small tomatoes) which of the healthy snacks they like most.

Energy expenditure related measures

Athletic competence. In addition to the general perceived competence scale (see PCS, Motivation) [60], specific self-perceived athletic competence is measured with the 10 item subscale from the Perceived Competence Scale for Children (PCSC) [71, 72] with response categories ranging from '*No, certainly do not*' (1) – '*Yes, certainly do*' (6). Example items are '*Are you good at sports?*' or '*Do you have confidence in doing new sports for the first time?*'.

Barriers to Physical Activity. Participant's perception of barriers to their PA are assessed with the 21 item Barriers to PA scale [73], which consists of 5 subscales: body-related, resources, social, fitness, and inconvenience barriers. Composite scores are calculated from the items pertaining to each subscale (barrier type). The barrier statements are prefaced by the main question '*How often do the following things prevent you from getting physical activity?*' and participants can choose a response on a 6-point scale ranging from '*Never*' (1) to '*Always*' (6). Example items are: '*I am self-conscious of my body when I do PA*' (body-related), '*I have too much homework*' (convenience), '*I do not have the skills*' (resource), '*I do not have anyone to do PA with me*' (social) and '*PA is too hard*' (fitness).

Daily activities and sports participation. Participant's daily activities that facilitate moderate to vigorous PA are assessed by asking on which days participants follow educational PA at school and go to a sports or dance facility outside school. In addition, participants are asked to indicate which sports they play from a list of most popular sports

played (e.g., soccer, hockey, tennis, volleyball, cycling, dancing, swimming, ice skating, etc.). They are allowed to check multiple sports and can add sports when they are not listed by clicking on the option '*other*.' The participants are also asked which type of physical activity they like most (e.g., cycling, dancing, walking the dog, playing soccer, etc.). Further, they are asked when they are the least physically active, followed by an open ended answering option.

Environmental factors. To assess environmental factors, participants are asked to indicate what type of environment they live in (response options: city, town or village) and what types of facilities are proximal to their home. Participants can select multiple options from a list [74], such as forest, parks, play grounds, grass, etc. They also have the option to add facilities if these are not included in the list.

Habitual physical activity. Participants are asked to recall PA (of yesterday) 3 times during each wave with items adapted from the Activity Questionnaire for Adolescents and Adults (AQuAA) [75], the Short QUestionnaire to ASsess Health-enhancing physical activity (SQUASH) and the 'Day in the life' Questionnaire adapted to assess Dutch habitual physical activity [76-78]. It assesses physical activity at school and during leisure time, active transportation to and from school and sedentary behaviors in leisure time. First, participants are asked whether yesterday was a school or weekend day, and if they were ill. When they indicated it was a school day, they are then asked to indicate a) how they went to school and b) home ('car/ bus/ bicycle/walking/other'), c and d) what they did most during their first and second break at school ('sedentary activity/standing/walking/running/no break'), e) what they did after school and before dinner, f) what they did after dinner (allowing multiple answering categories: watching TV/gaming/Internet/homework/chores/groceries or shopping/work/walking/outdoor activity/other, g) which sports they played in the afternoon and evening (open ended). When participants indicate that it was a weekend day or they were ill, they were then asked the same questions but the day was divided into morning, afternoon and evening (without any references to school).

Participants are also asked to recall how many days in the previous week they were physically active for at least 60 minutes [79]. In addition, participants are asked to indicate on a VAS scale ranging from 0 ('*nothing*') – 100 ('*very much*') how much they think they are physically active during the day as well as how competent they think they are.

Perceived physical activity and social norms. Participants are asked to estimate how many days a week they think people (parents and friends) in their social environment are active for at least 60 minutes (i.e., descriptive norm) and how many days they think they *ought* to be active according to their parents and friends (i.e., injunctive norm).

Injury check. Participants are also asked during each data collection wave whether they were injured.

Self-presentation motives. The Self-presentation Motives for Physical Activity Questionnaire (SMPAQ) [33] describes different motives for being physically (in)active, such as social goals or social anxiety. The SMPAQ also provides insights into a person's desire to control how he or she is perceived by others (i.e., impression management). This scale consists of 9 items. The first 5 items concern the 'acquisitive-agentic' domain and measure the motives of being physically active to acquire new achievements or mastery. The last 4 items concern the 'acquisitive-communal' domain, and measure the motives of being physically active to acquire interpersonal relationships and connections. The self-presentation statements are prefaced by the main statement '*I am physically active because my classmates...* ' and participants choose a response on a 6-point scale ranging from '*Completely disagree*' (1) to '*Completely agree*'(6). Example items are: '*...admire me for my physical ability*', '*...respect me for my physical ability*', '*....view me as physically fit when I exercise*' and '*...view me as kind*.'

(Social) Media consumption

Television (TV) exposure. Hours of TV watching is assessed by asking participants how many days per week they usually watch TV and for how many hours a day (ranging from '*Never*' (1), '*½ hour a day*' (2), '*1 hour a day*' (3), '*2 hours per day*' (4) to '*Seven or more hours a day*' (9) [80]. In addition, they are asked to indicate how often they usually watch TV in the morning, afternoon and evening, and how often they watch TV together with someone on a 6-point scale ranging from '*Never*' (1) – '*Always*' (6) [81]. Participants also indicate from 20 Dutch and non-Dutch TV stations (including *DisneyXD, Cartoon Network, National Geographic, Discovery Channel, MTV, TLC, Nickelodeon, 24Kitchen*) and *Netflix* whether and how frequently they watch it. Additionally, they are asked in an open-ended question (which was administered separately from the one described in the above) to estimate how many hours per day they usually watch TV.

Genre TV programs. The following items are included as 'filler items' to keep participation to the research project fun and the participants engaged. Nevertheless, these items provide interesting insights into the genres that Dutch youth watch. Participants are asked to indicate for each of the following genres how often they watch it on TV on a scale from '*Never*' (1) – '*Always*' (6): Sports, Cooking, Home improvement and Gardening,

Lifestyle, Talent Shows, Quizzes, Cartoons/Comedy, Reality TV, Soaps, Talk shows, News, Travel, Documentaries, Showbiz and Music.

Internet and social media exposure. Hours of Internet and social media use is assessed for the two behaviors separately, by asking participants how many days per week they usually use Internet and social media, and for how many hours a day (ranging from '*Never*'(1), '*½ hour a day*' (2), '*1 hour a day*' (3), '*2 hours per day*' (4) until '*Seven or more hours a day*' (9) [80]. In addition, they are asked to indicate for each of the behaviors how often they use it in the morning, afternoon and evening, and how often they use it on a 6-point scale ranging from '*Never*' (1) – '*Always*' (6) [81]. Participants also indicate which social media platform they use, which one is their favorite (e.g., *Facebook, Instagram, Snapchat, YoutTube, Pinterest, Vine, Tumblr, Twitter, Musical.ly, Google+, WeHeartIt, Messenger*), and how often they use the specific social medium on a 6-point scale ranging from '*Never*' (1) – '*Always*' (6). They could also add a social media platform and answer the frequency regarding the added platform. Additionally, they are asked in an open-ended format to estimate how many hours per day they usually use the Internet.

Video blog (Vlog) exposure. Participants are asked to indicate which 'Vloggers' they like (open-ended), how often they watch them on a 6-point scale ranging from '*Never*' (1) – '*Always*' (6) and which celebrities they follow on the Internet (open-ended).

Gaming behavior. Gaming is assessed by asking participants how many days per week they usually play games in general and on a game console specifically, and for how many hours each day (ranging from 'Never'(1), '½ hour a day' (2), '1 hour a day' (3), '2 hours per day' (4) until 'Seven or more hours a day' (9) [80]. They are also asked to indicate how often they gamed in the morning, afternoon and evening on a 6-point scale ranging from '*Never*' (1) – '*Always*' (6) [81]. In addition, they are asked how often they play games on different devices (i.e., smartphone, tablet, computer, game console), how often they played games on a game console together with someone, which one is their favorite and which one is most often played together. An open-ended item also asked to estimate how many hours per day they play games.

Genre games. The following items are assessed as 'filler items,' but provide interesting insights into the genres of videogames Dutch youth play. Participants are asked to indicate for each of the following genres how often they watch it on TV on a scale from '*Never*' (1) – '*Always*' (6): Action (e.g., *Call of Duty, Grant Theft Auto*), Adventure (e.g., *Minecraft, Skylanders*), Skills (e.g., *DumbWaysToDie, Angry Birds*), Puzzle, Word games, Racing, Sports, Music, Educational games (e.g., *Dutch Topography, Squla games*),

Simulation (e.g., *Sims, Goat simulator*), Exercise games (e.g., *Wii Sports, Kinect Sports Rivals*) and Virtual Reality (e.g., by using Virtual Reality glasses or *Oculus Rift*).

House rules on media use. Participants are asked about their caregivers' restrictions on their screen-based activities at home based on the revised scale of the Activity Support Scale for Multiple Groups (ACTS-MG) [82]. They are asked to indicate whether they were allowed as often as they liked to watch TV, use the computer for things other than homework (such as playing computer games and surfing the internet) and play video games (such as Playstation, Xbox, and Nintendo). Answers are administered on a scale ranging from '*No, certainly do not*' (1) – '*Yes, certainly do*' (6).

Mealtime media use. The frequency of participants' use of electronic media (e.g., tablet use, talk or send messages on their phone, watch TV, listen to music or play games during mealtime) is described with 6 items on a 6-point scale ranging from '*Never*' (1) – '*Always*' (6) [83].

Use of health apps and Wearables. Participants are asked how much they like health apps (i.e., apps about healthy eating and drinking and apps about sports and physical activity) on a 4-point scale ranging from '*Do not like it at all*' (1) to '*Like it a lot*' (4), how often they use it on a 6-point scale ranging from '*Never*' (1) – '*Always*' (6), which one(s) they use, and what they like about them (followed by an open ended answering option). In addition, they are asked about the apps' effectiveness (e.g., whether they think it would influence their behavior) on a scale ranging from '*No, certainly do not*' (1) – '*Yes, certainly do*' (6), and whether they have downloaded the app(s) themselves ('*Yes*' or '*No*').

In addition, questions are administered about whether participants like Wearables and think it stimulates them to be active. They are also asked whether they have their own activity tracker and if they do, they are asked which type they own, how often they use it, and what they like about it.

Research and app evaluation

Evaluation research and app. The app evaluation provides useful feedback about the user friendliness of the app, avatar, game, *Social Buzz*, etc. On the last day of the data collection period, participants are asked how they liked participating in the *MyMovez* research in general, answering daily questionnaires, wearing the activity tracker wristband, how much they like the app, the game, the avatar, jokes and memes, and the *Social Buzz* (in wave 4). They answered about each aspect on a 4-point scale ranging from '*Do not like it at all*' (1) to '*Like it a lot*' (4). In addition, they are asked to indicate what they like and do not like about

the app, how their parent(s) and teacher(s) feel about their participation, whether they always took the smartphone with them (‘*Yes*’ or ‘*No*’, and if not, this is followed by an open question to explain why), how often they answered questions (on a 6-point scale ranging from ‘*Never*’ (1) – ‘*Always*’ (6)), whether the smartphone was easy to use (on a 4-point scale ranging from ‘*No, absolutely not*’ (1) – ‘*Yes, absolutely sure* (4), and whether they succeeded in charging the phone (‘*Yes*’ or ‘*No*’, and if not, followed by an open question to explain why). In wave 4, they are also asked how often they use the Social Buzz in general, to just read posts of others, and to post messages themselves (‘*Never*’ (1) – ‘*Always*’ (6)). In addition, they can indicate which folder containing pictures to send around (e.g., of animals, sports, food, etc.) they liked most, and which picture was their favorite to spread and receive, whether they liked the group Buzz or 1-on-1 Buzz, and which one they used most frequently. Finally, they are asked to rate the *MyMovez* app on a VAS scale ranging from 1 to 10.

Enjoyment participation MyMovez. Enjoyment in participating in the MyMovez research is measured on a 6 item scale, based on the Intrinsic Motivation Inventory [61]. The scale measures participant’s liking, enjoyment and feeling of pleasure to participate (e.g., *Do you like participating in the MyMovez research?*’) with answer options ranging from ‘*I completely disagree*’ (1) – ‘*I completely agree*’ (6).

Filler items

Filler items are used to keep participants engaged and to make the research more attractive and fun. Filler items include naming their favorite foods, music, brands, animals, movies, celebrities, sports and sports heroes, daily planning, summer holiday plans, knowledge of several subjects (e.g., fashion, celebrities, politics, sports, games) and enjoyment of school, which were all rated on a VAS scale from 0 to 10. Other filler items concerned jokes, memes and riddles or asking them to fantasize about what they would do when being invisible or what they would name their own island.
